# Levels and Predictors of Leaders' Humble Leadership, Participants' Psychological Safety, Knowledge Sharing in the Team, and Followers' Creativity in Nursing: A Cross-Sectional Online Survey

**DOI:** 10.1155/2024/9660787

**Published:** 2024-06-12

**Authors:** Majd T. Mrayyan, Saleem F. Al-Rjoub

**Affiliations:** Department of Community and Mental Health Nursing, Faculty of Nursing, The Hashemite University, P.O. Box 330127, Zarqa 13133, Jordan

## Abstract

**Aim:**

The current study investigated the levels and predictors of leaders' humble leadership, participants' psychological safety, knowledge sharing in the team, and followers' creativity in nursing.

**Background:**

Humble leadership, psychological safety, knowledge sharing, and followers' creativity are non-nursing research fields, and humble leadership has recently been examined in nursing.

**Methods:**

A cross-sectional research design was employed via an online survey. A nonprobability convenience snowball sample of 245 nursing academics (*n* = 85, 34.70%), nurses (*n* = 140, 57.10%), and nursing leaders (*n* = 20, 8.20%) was recruited from three universities and three hospitals.

**Results:**

The participants rated “high” the leaders' humble leadership, knowledge sharing in the team, and followers' creativity in nursing. However, participants' psychological safety was precarious. The four variables' predictors were assessed based on the sample's characteristics. Leaders' humble leadership did not predict participants' psychological safety; the sole predictor of the variable was the organization's quality initiatives. The predictors of knowledge sharing in the team were leaders' humble leadership, age, level of education, and accreditation initiatives in the organizations. The predictors of followers' creativity were leaders' humble leadership, level of education, and quality initiatives in the organizations. The lowest means of the four variables should be immediately managed.

**Conclusion:**

Quality initiatives in organizations and the number of tenures were the most influential predictors of the four variables evaluated. Leaders' humble leadership predicted knowledge sharing in the team and followers' creativity, but not participants' psychological safety. As followers' psychological safety contributes to trustful relationships within the team, workplace boundaries and conducive work environments should be promoted. Training programs are required to develop humble nurses and leaders' leadership.

## 1. Background

Despite the developments in nursing leadership research, humble leadership continues to have certain evident disadvantages [1, 2]. Social information processing theory provides the theoretical basis for understanding how humble leadership influences followers' creativity [3]. This theory suggests that followers rely on information cues to understand their work environments and regulate their behaviors [3, 4]. Leaders are the key information sources, given their higher status and direct involvement and interactions with followers [5, 6].

Today's healthcare environment faces many challenges that include but are not limited to development and innovations, meeting international requirements and competition, and developing new leadership approaches, such as humble leadership [7].

This leadership style is a new concept that has gained more attention in nursing leadership research as the term originates originally in psychology research [6, 7]. Humble leadership, also called humility, is a top-down leadership style that focuses on leaders' openness about their developmental processes, including willingness to view themselves accurately, being open to feedback and creative ideas, and learning by doing [6–8]. This style is needed in nursing because nursing leadership has vital roles in healthcare services at all levels [7]. Nursing humble leaders have to create conducive work environments that promote nurses' engagement, creativity, and innovation [7] and, in turn, achieve different healthcare outcomes, particularly patient satisfaction and the quality of nursing care. An example of these outcomes is missed care or rationed care, which is a highly prevalent clinical healthcare problem resulting in many consequences that negatively influence patients, nurses, and healthcare organizations [9]. Organizations that have quality initiatives have more humble leaders [10], who are expected to solve efficiently the complicated issue of missed care by employing different interventions such as appropriate staffing levels, supplying enough resources, mutual communication and feedback, and teamwork [11].

Psychology-related variables include humble leadership, psychological safety, knowledge sharing, and followers' creativity. Traditional leadership is no longer appropriate because it is difficult for today's leaders to figure everything out at the top due to the increasingly chaotic healthcare work environment [6, 8]. In the quickly changing work environment, nurses' creativity and vigilant leaders have become a mandate [12, 13]. Nevertheless, the growth of creativity is not an instantaneous process; it necessitates an environment of psychological security and the exchange of knowledge to thrive [13, 14].

Being open to new ideas and criticism, valuing the abilities and contributions of others, and seeing oneself honestly are all interpersonal traits of *humble leadership* [15, 16]. Leadership researchers have only recently begun to empirically investigate the impact of leaders' humble leadership on followers' attitudes, such as job satisfaction and job engagement [16], psychological empowerment [6, 13, 15], and nurses' innovative behaviors [15, 17]. A humble leader's behavioral modeling A leader's humble approach can foster a mentally secure setting, potentially shaping a psychologically safe environment [15, 17], which, in turn, may inspire followers to engage in creative behaviors [6, 15].

Psychological safety is the perception of the consequences when taking interpersonal risks in work environments [6, 15]. For followers to be creative, a sense of safety is required [6, 10]. Humble leadership significantly impacts psychological safety [6, 14, 15]. Humble leaders accept their shortcomings and errors in public and see mistakes as a valuable part of the learning process [15, 18]. Such behaviors provide critical information to followers, allowing them to feel psychologically comfortable expressing themselves and taking interpersonal risks to realize their full potential, which also applies to nursing professionals. This is especially true when followers of humble leaders form remarkable leader-follower relationships [15, 16] as it lowers perceived risks and strengthens the safety climate [6, 15].

In addition, humble leaders constantly seek feedback and are open to new ideas [15]. This communicates to followers that speaking up and expressing novel ideas are acceptable and even expected [6, 15]. Humble nursing leaders appreciate their team members' talents and efforts [10] and foster a psychologically safe environment [19] by valuing and encouraging their ideas and opinions [6, 14]. As a result, the followers (nurses) feel free to express themselves and work without fear of negative consequences [6, 19].

Organizations must be creative to perform well, survive, and succeed [20, 21]. The value of creativity has boosted research interest in uncovering traits that lead to creativity during the last few decades [6]. Most of these studies emphasized the positive benefits of leadership styles, such as authentic, transformational, servant, empowering, and sharing, which can positively influence followers' (nurses') creativity [6, 21].

Creativity is inherently riddled with obstacles, uncertainties, and risks because new ideas cannot be guaranteed to produce the desired results [22]. In addition, leaders may not always support or accept new ideas from followers [6, 23]. As a result, a work environment that supports interpersonal risk-taking and expressing novel ideas is critical for motivating followers' creativity; it can even increase one's propensity to do so [6, 24]. Individuals are more likely to express new ideas and take risks in a safe environment, which helps them overcome their anxiety and fear of failure [6, 25].

The psychological safety of the followers has influenced their creativity [6, 14]. However, research on the connection between psychological safety and creativity has yielded contradictory results [6]. Surprisingly, psychological safety does not always result in creativity [6, 26]. As a result, the relationship between psychological safety and creativity is more complicated than anticipated [6, 27]. According to inconsistent empirical findings, boundary variables permit or inhibit the favorable impacts of psychological safety on creativity [6, 28]. However, regardless of the contradictory findings, a psychologically safe environment fosters intergroup trust [13], allowing followers to engage in risky creative activities [6, 14, 25]. Knowledge sharing is crucial for participants' psychological safety [14, 25] and creativity and motivation [12, 28]. The absence of cognitive capacity among followers hinders the generation and presentation of creative ideas [6, 29].

Knowledge sharing is essential for followers' creativity [6, 14]. Knowledge sharing allows followers to gain cognitive resources such as ideas, knowledge, and information, improve cognitive capabilities, and inspire creativity [6, 12, 14].

Research problem: due to the constantly changing work environment, today's leaders find it difficult to grasp humble leadership at the top [15, 18]. Top-down leadership has gone out of step with the times [30]. Despite calls for humble leadership, many unknowns exist regarding how it works in organizations, including healthcare and nursing [6, 18, 19].

As a result of their leaders' higher status and frequent interactions with their teams, followers often learn important information from their leaders' comments and behaviors, allowing them to alter their perspectives of work settings and respond in the desired way [6, 31]. When humble leaders recognize and manage their flaws and errors, value their followers' strengths and contributions, and display teachability, followers may feel psychologically safe to speak and share new ideas [18, 32]. In response, the perception of a safe work environment may enhance followers' creativity [6, 33].

Theoretical background: despite the importance of humble leadership, there is a significant gap in knowing how humble leadership works in organizations [6, 18]. Consistent with Wang et al. [6] and Elhadidy and Gao [3], “social information processing” theory was adopted in this study. That is, leaders should give up the concept of the “great man,” be transparent about their knowledge and experience limitations, and pay closer attention to the influence of followers on leader's performance. As a result, this study investigated how humble leadership influences followers' creativity.

The social information processing theory focuses on how individuals use environmental information to build and interpret events in the workplace [3, 4]. Followers seek information from their leaders to shape their perceptions of the work environments [6, 34]. They act as per the situational attractiveness of specific activities [6]. We assume that humble leaders admit their faults and shortcomings, demonstrate teachability, and value the abilities and contributions of their followers [18, 19]. Followers will feel psychologically comfortable to express fresh thoughts in this circumstance [6, 33]. As a result, followers' creativity would be encouraged if they perceived a safe work environment [6, 33]. According to social information processing theory, psychological safety contributes favorably to modest leadership and followers' creativity [3].

Purpose and significance: nursing research on humble leadership, psychological safety, knowledge sharing in the team, and followers' creativity is lacking. Like many other countries, Jordan is experiencing volatile work situations; hence, such a study is timely.

This is a large-scale project in which the current study answered the following research questions: (1) what are the levels of leaders' humble leadership, participants' psychological safety, knowledge sharing in the team, and followers' creativity in nursing? (2) What are the predictors of leaders' humble leadership, participants' psychological safety, knowledge sharing in the team, and followers' creativity in nursing based on the subjects' characteristics? (3) Do the leaders' humble leadership and the subjects' characteristics predict participants' psychological safety, knowledge sharing in the team, and followers' creativity in nursing?

Only one psychology-based study studied humble leadership, psychological safety, knowledge sharing, and followers' creativity together [6]. Our study is the first international and national study about the four concepts in nursing. Some studies exist about some concepts but were not conducted in nursing [35] or had limited concepts in nursing studies, such as leaders' humble leadership and nurses' psychological safety [19].

The outcomes of the current study will be used to develop leadership training for nurses and other professionals. Nursing leaders should create supportive work environments to encourage followers' psychological safety and cultivate trusting relationships with their leaders and other experts. Leaders should share knowledge to help their team members achieve psychological safety because it has been demonstrated to reduce the impact of participants' psychological safety on their creativity. Organizations that foster psychological safety and knowledge-sharing circumstances should see high levels of followers' creativity.

## 2. Methods

### 2.1. Research Design and Sampling Technique

An online survey was used with a cross-sectional design. A nonprobability convenience snowball sample of 245 nursing academics (*n* = 85, 34.70%), nurses (*n* = 140, 57.10%), and nursing leaders (*n* = 20, 8.20%) who worked in three universities and three hospitals were collected, with a response rate of 70.00%. Facebook and WhatsApp were used to collect responses from participants for the online survey. The principal investigator started collecting data by recruiting convenience samples through which the study was announced, and participants were selected if they wished to participate. Other participants were referred to the researcher through snowball recruitment.

The sample size in this study was determined by the formula of *N* ≥ 10(k) + 50, where N is the sample size and *k* is the number of independent variables (four major variables and 12 subjects' characteristics); the minimum sample size should be 210 participants [36], however, 245 participants were obtained. The ability to use electronic platforms and being employed in universities or hospital settings were the sample inclusion criteria.

### 2.2. Ethical Considerations

The university's Institutional Review Board (IRB), where the current authors work, approved the study; the reference number is 11/8/2021/2022, dated July 25, 2022. Participants were informed in the invitation letter that they might decline to complete the online survey if desired and that their participation constituted their consent. The researchers stored a password on participants' coded responses in Google Drive; all participant data were anonymous. Confidentiality was assured by sharing the aggregate results with nursing leaders in the designated universities and hospitals.

### 2.3. Data Collection Procedures

Data were gathered in 2022 using a self-report survey administered online in English using Google Forms. The online survey was set to allow for one submission only and by using cookies. It was validated through a small pilot study to check that it was functional and appropriate for the Jordanian setting; no changes were required. The first researcher shared the survey link on the Facebook pages of the Faculty of Nursing and the first researcher's colleagues. To ensure that the target population of nursing academics, nurses, and nursing leaders was targeted, filtering questions were used at the beginning of the survey. The participants were encouraged to invite their contacts. Data were collected across ten days, and participants were reminded to complete the survey only once after five days with a reminder e-mail. As a result, the online survey was built in this manner.

### 2.4. Research Instruments: Variables and their Operational Definitions

The subjects' characteristics measured in the current study were gender, marital status, age, time commitment, level of education, and work area if the organization of work is being accredited or if they have official quality initiatives (yes vs. no), tenure (a tenured is someone who has worked for an organization for several years), team size, type of sector, and title.

Following the methodology of Wang et al. [6], the English-version tools were used to collect data and piloted before the actual data collection; no adjustments were required. These research instruments are commonly used in psychology because they have high psychometric measures. Humble leadership, psychological safety, knowledge sharing, and creativity were continuous variables. All tool is a five-point Likert scale ranging from 1 (strongly disagree) to 5 (strongly agree). Thus, a score above 3 to 5 was considered high; around three was considered edgy or borderline, and below three was low.

#### 2.4.1. Humble Leadership

The nine-item scale developed by Owens et al. [16] was used to measure leaders' humble leadership. “This leader often compliments others strength” is an example of an item. The original instrument has content and incremental validity that differentiate it from other positive leadership styles, such as servant, transformational, and ethical leadership [2]. The Cronbach's alpha for this instrument was 0.88 [6] and 0.93 in the current study.

#### 2.4.2. Psychological Safety

The seven-item scale Edmondson [37] developed was used to measure participants' psychological safety. “It is difficult to ask other team members of the organization for help” is an example. The original instrument has discriminant validity [37]. The Cronbach's alpha for this tool was 0.76 [6] and 0.71 in the current study.

#### 2.4.3. Knowledge Sharing

The seven-item scale developed by Lee [38] was used to measure knowledge sharing in the team. “Organizational members share each other's success and failure stories” is an example of an item. The original instrument was reported to have predictive validity [39]. The Cronbach's alpha for this instrument was 0.78 [6] and 0.93 in the current study.

#### 2.4.4. Creativity

The 12-item self-rated creativity scale (SRCS) was modified [40] and used to measure followers' (nursing academics, nurses, and nursing leaders) creativity. “I come up with new and practical ideas to improve performance” is an example. The SRCS was created to evaluate employees' creativity [40]. The original instrument was reported to have convergent, discriminant, and concurrent validities [40]. The SRCS had internal consistency, as indicated by the Cronbach's alpha of 0.84 [41] and 0.94 in the current study.

### 2.5. Data Analyses

The Statistical Package for the Social Sciences (SPSS) version 25 [42] calculated descriptive statistics as means, standard deviations, or frequencies and percentages at an alpha of 0.05. Data were visually checked for normality and outliers by drawing a histogram and checking for extreme standard deviations. There were no missing data as the online survey was set to answer all questions.

For the current study, humble leadership and subjects' characteristics were treated as independent variables, while psychological safety, knowledge sharing, and followers' creativity were the dependent variables.

The general linear model (GLM) was used to assess predictors of leaders' humble leadership, participants' psychological safety, knowledge sharing in the team, and followers' creativity based on subjects' characteristics [36]. Another GLM was run while assigning leaders' humble leadership and subjects' characteristics as independent variables, and participants' psychological safety, knowledge sharing in the team, and followers' creativity as dependent variables [36].

Following previous research [6], this study includes several control variables. The first researcher controlled gender, age, the number of tenures (the duration a person holds a job or title), and team size at the organization. Followers' gender, age, and team size were considered because they relate to followers' creativity [6].

## 3. Results

The majority of the sample was married females, nursing academics, or nurses or nursing leaders, young to middle-aged with baccalaureate degrees, and full-time nurses or academics; these governmental work settings were mostly accredited and had quality initiatives. The participants reported that they were tenured for ten years or more (above average tenure; the average is commonly four years) and worked in a large team with more than fifteen members (the ideal collaborative team size is 4–8) (Table 1).

### 3.1. Levels of Leaders' Humble Leadership

For the whole sample and a five-point Likert scale, the participants rated highly (agreed) the leaders' humble leadership (mean = 3.57; SD = 0.87). The highest mean of leaders' humble leadership was that the leader was open to the ideas of others (mean = 3.72; SD = 1.06) and showed appreciation for the unique contributions of others (mean = 3.71; SD = 1.06). The lowest mean of leaders' humble leadership was that they admitted when they did not know how to do something (mean = 3.28; SD = 1.14) (Table 2).

### 3.2. Levels of Participants' Psychological Safety

For the whole sample and a five-point Likert scale, the participants' psychological safety was on the border (mean = 3.09; SD = 0.64). The highest mean of participants' psychological safety was that they felt their unique skills and talents were valued and utilized when working with team members (mean = 3.49; SD = 0.98). The lowest mean of participants' psychological safety was having difficulty asking others for help (mean = 2.71; SD = 1.15) (Table 3).

### 3.3. Levels of Knowledge Sharing in the Team

For the whole sample and a five-point Likert scale, the participants rated high (agreed) knowledge sharing in the team (mean = 3.48; SD = 0.80). The highest mean was related to the implicit knowledge sharing related to service providers sharing “know-how” from work experience with each other (mean = 3.60; SD = 0.88). The lowest means were related to the explicit knowledge sharing related to service providers sharing each other's success and failure stories (mean = 3.42; SD = 1.01) and business knowledge obtained from different sources (mean = 3.42; SD = 1.01) (Table 4).

### 3.4. Levels of Followers' Creativity

For the whole sample and a five-point Likert scale, the participants rated high (agreed) followers' creativity within the team (mean = 3.75; SD = 0.63). The highest mean of followers' creativity was that they suggested new ways to increase the quality of work (mean = 3.90; SD = 0.92). The lowest mean of followers' creativity was that they were not afraid to take risks (mean = 3.59; SD = 0.95) (Table 5).

### 3.5. Significant Predictors of Leaders' Humble Leadership, Participants' Psychological Safety, Knowledge Sharing in the Team, and Followers' Creativity Based on the Subject's Characteristics

The GLM was used to assess predictors of the studied variables based on the subject's characteristics [36].

The predictors of leaders' humble leadership were marital status (*t*-test = −2.75; *p* value = 0.006) and the number of tenures (*t*-test = 1.26; *p* value = 0.010). The model was significant (F-test = 16.88; DF = 12; *p* value = 0.001; adjusted *R*^2^ = 0.037) and explained 3.7% of the variance in the mean score of leaders' humble leadership.

The predictor of participants' psychological safety was only the quality initiatives in the organizations (*t*-test = −2.37; *p* value = 0.018). The model was significant (F-test = 18.78; DF = 12; *p* value = 0.001; adjusted *R*^2^ = 0.012) and explained 1.2% of the variance in the mean score of participants' psychological safety.

The predictors of knowledge sharing in the team were marital status (*t*-test = −2.87; *p* value = 0.004), the accreditation initiatives in the organizations (*t*-test = −2.79; *p* value = 0.006), the quality initiatives in the organizations (*t*-test = −2.22; *p* value = 0.027), and the number of tenures (*t*-test = 2.39; *p* value = 0.018). The model was significant (F-test = 16.74; DF = 12; *p* value = 0.001; adjusted *R*^2^ = 0.103) and explained 10.3% of the variance in the team's mean score of knowledge sharing in the team.

The predictors of followers' creativity were the level of education (*t*-test = 2.70; *p* value = 0.007), the quality initiatives in the organizations (*t*-test = −3.93; *p* value = 0.001), and the number of tenures (*t*-test = 2.20; *p* value = 0.029). The model was significant (*F*-test = 11.69; DF = 12; *p* value = 0.001, adjusted *R*^2^ = 0.133) and explained 13.3% of the variance in the mean score of followers' creativity (Table 6).

### 3.6. Leaders' Humble Leadership and Subject's Characteristics as Predictors of Participants' Psychological Safety, Knowledge Sharing in the Team, and Followers' Creativity

The GLM was performed while handling leaders' humble leadership and subjects' characteristics as independent variables, and participants' psychological safety, knowledge sharing in the team, and followers' creativity as dependent variables [36].

The results of the GLM indicated that the only predictor of participants' psychological safety was the quality initiatives in the organizations (*B* = −2.16; *p* value = 0.020). The model was significant (*F*(df = 13) = 17.11; *p* value = 0.001; *R*^2^ = 0.008; Table 7), and it explained 0.80% of the variance in the mean score of participants' psychological safety.

The predictors of knowledge sharing in the team were leaders' humble leadership (*B* = 8.16; *p* value = 0.001), age (*B* = 2.79; *p* value = 0.006), level of education (*B* = −1.99; *p* value = 0.047), and the accreditation initiatives in the organizations (*B* = −2.55; *p* value = 0.011). The model was significant (F(df = 13) = 5.51; *p* value = 0.020; adjusted *R*^2^ = 0.301; Table 7) and explained 30.10% of the variance in the team's mean score of knowledge sharing.

The predictors of followers' creativity were leaders' humble leadership (*B* = 7.16; *p* value = 0.001), level of education (*B* = 2.78; *p* value = 0.006), and the quality initiatives in the organizations (*B* = −3.57; *p* value = 0.001). The model was insignificant (F(df = 13) = 3.15; *p* value = 0.077; Table 7), but it explained 28.70% of the variance in the mean score of followers' creativity.

In conclusion, the most influential predictors of leaders' humble leadership, participants' psychological safety, knowledge sharing in the team, and followers' creativity were quality initiatives in the organizations and the number of tenures. Leaders' humble leadership predicted knowledge sharing in the team and followers' creativity, but not participants' psychological safety.

## 4. Discussion

The current study's subjects fit the demographics of nursing academics, nurses, nursing leaders, and organizational characteristics in Jordan.

According to the social information processing theory [3, 4], humble leadership influences followers' creativity when they are well-informed, and knowledge is shared among the team; this reflects positively in their understanding of their work environments and regulating their behaviors accordingly [3, 4]. However, the staff's need for self-monitoring and constant scanning of their work environments may explain why they have borderline psychological safety.

The high rating for humble leadership stems from the benefits of humble leadership, which pave the path for better listening and improved collaboration (supported by Anser et al. [43] and Kelemen et al. [15]). As former nurses, nursing leaders, and current academic professionals, we like to be led by a sympathetic leader.

The high rating for knowledge sharing emanated from knowledge being one of any organization's most valuable intangible assets. While corroborating the assertions mentioned above as former healthcare professionals and current academics, Al Hawamdeh [44] asserted that staff members have grown more receptive to knowledge help. Staff employees are more prepared to deal with unexpected events since they are more knowledgeable. Thus, the study participants were aware that knowledge would flow from top leadership to staff members through knowledge exchange in the team, thus setting the way for creating relevant or new knowledge in the organization.

Followers' creativity within the team received a higher rating because of the improved teamwork it delivers to an organization, which is consistent with the studies of Mrayyan [10], Sengupta et al. [45], as well as Wang et al. [6]. Collaboration is widely encouraged in a creative environment. As a result, the participants realized they needed to foster a continuous learning attitude while encouraging employees to seek new information, knowledge, and better ways to complete their responsibilities.

However, despite all preceding positive indicators, participants' psychological safety was borderline; this may imply issues with inclusion in teams and organizations, as Singh [14] suggests. In Jordan, we suffer limited inclusion in teams and organizations. Psychological safety is about enabling openness, inclusion is necessary for mutual learning, and the latter is necessary to progress in a complex and ambiguous world [46]. This pioneer researcher in psychological safety attested to the strong link between psychological safety, learning, and performance in teams and organizations.

### 4.1. Leaders' Humble Leadership

The participants rated highly (agreed) the leaders' humble leadership. This point again humbles leadership to leaders' ability to recognize their strengths and weaknesses alongside limitations, similar to Cho et al. [47] and Kelemen et al. [15].

Our participants agreed that a humble leader can be more persuasive [48]; this is human nature. Humans frequently support the underdog while opposing their competitors or opponents. Humble leadership puts leaders in a better position to inspire their team or followers. Furthermore, nurse leaders may readily persuade their followers to collaborate to realize a healthcare vision. Humble leadership engenders trust, loyalty, and enthusiasm more effectively than fear and manipulation, which is in line with studies of Kelemen et al. [15].

Humble leaders are open to other people's ideas and show respect for their unique contributions. This was the polar reverse of popular stereotypes, calling for nurse leadership to command a room with high success and authority. Participants recognized the importance of adopting a more inclusive and inviting communication style. The participants were part of a nursing leadership that encouraged workers to discuss their ideas. Taking this option provides high operational flexibility and considerable levels of employee involvement. In comparison to their competitors that embrace authoritarian techniques, such nursing organizations demonstrate strategic alignment and retention, as well as a high level of performance [13], which is supported by Kelemen et al. [15].

On the other hand, humble leaders did not admit when they did not know how to do something. This illustrates that one of the major characteristics of humble leadership is the willingness to be taught and directed in areas where they are either inexperienced or lack knowledge. This kind of admittance shows the nurses their leaders and also fix opinion as opinions, and it helps the team come up with the right solutions, as demonstrated by Zhou and Li [49].

Some nurses may have encountered something similar in past work contexts, possibly in other roles. As a result, they will provide vital insight into the next best action. As seen across leading healthcare organizations, the most significant successes will likely emerge from not knowing [49].

### 4.2. Participants' Psychological Safety

The participants rated their psychological safety on the border, contrary to Wang et al. [6] and similar to Mrayyan and Al-Rjoub [19]; this warrants vigilant leadership to intervene immediately. Psychologically safe staff members, on the other hand, were open to ideas that would eventually lead the way for new solutions, as demonstrated by O'donovan and Mcauliffe [50]. Nursing organizations acknowledge the power of variety through psychological safety. As a result, the participants advocate for psychologically safer workplaces throughout their businesses, with nurses encouraged to suggest new and innovative solutions.

Participants' psychological safety was high when they felt their unique skills and talents were valued and utilized when working with team members. This could imply that nursing leadership can provide psychological safety to team members by exploiting their particular abilities and talents. This will demonstrate to them that their organizations value their abilities and capabilities. The findings align with Mansour and Tremblay [51], who claimed that when firms better utilize their employees' skills, they have a higher job satisfaction. Nursing staff workers are more prepared to adjust to recommended future changes when they have psychological safety.

On the other hand, participants' psychological safety was low when they had trouble asking others for support. Taylor et al. [52] validated the findings, arguing that some professionals, even leaders, think that asking for help will expose them as inexperienced or incapable. The researchers reported that some nursing staff members think that such a request may be a process of losing control. In many organizations, such as ours, asking for help might put the staff member in a vulnerable position, where the majority may avoid seeming weak, which is similar to the study of Taylor et al. [52].

### 4.3. Knowledge Sharing in the Team

The participants rated high (agreed) knowledge sharing in the team. Knowledge sharing emerged as a crucial attribute of nursing leadership, as did humble leadership [6, 10, 14]. Confirming our findings with Holmgren et al. [53], the participants recognized the importance of knowledge sharing in increasing the collective skills of medical staff members. Understanding a patient's condition and making healthcare-related decisions demonstrate the capacity.

The highest level of implicit knowledge sharing was associated with service providers exchanging “know-how” from their job experiences with one another. This step is crucial in ensuring that the team connects and performs better [6, 54]. Similarly to our view, Holmgren et al. [53] suggested that sharing implicit knowledge of the experience commanded by other nurses helps them become more vital professionals. This allows the organization to save more training resources while still capturing and maintaining knowledge during turnover [53].

The least explicit information sharing occurred when service providers shared each other's success and failure tales and business expertise collected from various sources. Participants agreed that, similar to our professional experience, such a move was critical in cooperating and producing collective knowledge. Explicit knowledge sharing promotes improved ways of carrying out nursing duties while fostering a learning culture [53].

### 4.4. Followers' Creativity

The participants rated high (agreed) followers' creativity within the team. The participants were aware that creativity is a factor that inspires staff members to operate as a team, as attested by Kohnen et al. [34]. As a result of such a creative process, an organization fosters a collaborative culture. Thus, with a continuous learning mindset, staff members would continuously seek new information and expertise as well as better ways of doing things [43].

When followers offered new approaches to improve the quality of work, their creativity was high. Creativity encompasses increased quality and quantity production [43]. As mentioned, staff members realize this when seeking new information and knowledge, which is possible when they identify new ways to do their jobs, as previously said.

Participants were unafraid to take risks, indicating that creative individuals are risk-takers. Creative people are always ready to take “good” risks, which leads them down an inventive road [43]. Furthermore, they will take social risks to keep the team safe.

### 4.5. Significant Predictors of the Studied Concepts Based on the Subject's Characteristics

The predictors of leaders' humble leadership were marital status and the number of tenures, which is one of the novel results of the current study. The current findings are supported by Wang et al. [55], who reported that the antecedents of creativity in teams were leaders' gender, marital status, age, education, and tenure, which may also apply to our leaders' humble leadership. Being female and having more tenure years may signify having better job control and self-discipline, as mentioned by Al Hawamdeh [44]. Similar to our situation, the researcher reported that gender and tenure made a leader more caring while exhibiting high levels of self-control. As a result, the staff members were more focused on achieving personal goals and meeting organizational objectives.

The predictor of participants' psychological safety was only the quality initiatives in the organizations, which is also one of the novel results in the current study. As Jordan is experiencing quality initiatives in most clinical and academic settings, organizational quality initiatives promote our belief that the nursing team enjoys the safety necessary for interpersonal risk-taking. Quality initiatives are critical in creating a psychologically safe environment where people can speak up and express their views.

Marital status, accreditation initiatives in organizations, quality initiatives in organizations, and the number of tenures were predictors of knowledge sharing in the team, which is a novel finding. The four variables may contribute to our participants' trust in knowledge sharing. Being female and having tenure were antecedents of team creativity, supported by Wang et al. [55]. This may also apply to knowledge sharing in our teams in the current study. Chen et al. [1] complemented our findings about the effect of accreditation and quality initiatives in organizations on team knowledge sharing. The researchers asserted that staff members working in accredited organizations confidently share their knowledge. The same applies to the quality initiatives in organizations that always keep the staff members' input and output in check.

Of the novel results, the predictors of followers' creativity were the level of education, the quality initiatives in the organizations, and the number of tenures. This demonstrates that creative people are more educated and experienced. Besides, with quality initiatives in place, staff members would always seek new ways of doing things, similar to what is reported by Chen et al. [1].

### 4.6. Leaders' Humble Leadership and Subject's Characteristics as Predictors of the Studied Concepts

The only predictor of participants' psychological safety was the quality initiatives in the organizations. This result is in line with our participants' rating high (agreed) the leaders' humble leadership (similar to Mrayyan and Al-Rjoub [19] and Wang et al. [6]), but not their psychological safety (similar to Li et al. [26] and Mrayyan and Al-Rjoub [19]).

Previous studies reported a mediational model of psychological safety in the relationship between leadership and followers' creativity to account for the inconsistent findings in the literature [6, 26]. Although Wang et al. [6] reported that psychological safety has a mediating role between leadership and followers' creativity, Li et al. [26] reported no evidence of psychological safety mediation.

Previous research supported the relationship between leaders' humble leadership and participants' psychological safety [6, 56]; however, our study revealed that leaders' humble leadership does not predict participants' psychological safety. As former nurses and current academics, we strongly believe the problem is a lack of workplace boundaries. As a result, humble leaders must establish boundaries necessary for participants' psychological safety. Boundaries in the workplace are guidelines that employees make for themselves to determine what is psychologically safe and permissible for others to do around them [57]. Since they did not care for their well-being, our participants did not believe their workplace boundaries were healthy. Their team relationships did not blossom or grow well. They lacked the trust and belonging required to participate in the team. Everyone wants to set boundaries and have those boundaries respected. Healthy workplace boundaries are promoted by healthy work environments and cultures [57]. On the other hand, unhealthy boundaries lead to disengagement, depression, stress, and anxiety, and, this in turn, results in stress-induced physical illness [57].

Leaders' humble leadership, age, education, and organization accreditation initiatives were predictors of knowledge sharing in the team, which were comparable to Wang et al. [6], who reported that humble leadership influences knowledge sharing. Knowledge sharing is a humble leadership-boundary factor to enhance followers' creativity [6, 58]. Our participants rated high their humble leaders and knowledge sharing in the team. When there is a high level of knowledge sharing in the team, followers will offer and share new ideas, which does not suddenly happen. According to Mrayyan [10] and Wang et al. [6], humility leadership is the defining factor in such a setting and humble leaders should encourage team members to offer new ideas.

Leaders' humble leadership, education, and organizational quality initiatives were predictors of followers' creativity. According to Elhadidy and Gao [3] and Wang et al. [6], humble leaders influence their followers' creativity. These humble leaders were receptive to other people's views and appreciated the distinctive contributions of others [18, 19]. Thus, these positive characteristics of humble leaders will influence followers' creativity.

Chaman et al. [12] explained the psychological mechanisms between leaders' humble leadership and followers' creativity through intrinsic motivation and social learning. Those studies, however, concentrated on the old top-down leadership [30], whereas humble leadership is a new bottom-up leadership style [8]. Humble leaders are essential for any organization; however, humble leadership has only recently begun [59]. Moreover, Wang et al. [6] critiqued these studies for focusing just only on one level of analysis, either the individual level or the team level. This single level of analysis resulted in an incomplete picture of what humble leadership is, implying that humble leadership has cross-level effects, or what Li et al. [26] referred to as the “spillover” from “team-level humble leadership” to “individual-level humble leadership.”

### 4.7. Limitations

When nurses reflect on their leaders' humble leadership, they focus solely on their strengths, creating a bias in rating humble leaders. Self-reporting of leaders' humble leadership, participants' psychological safety, knowledge sharing in the team, and followers' creativity in nursing suggest possible bias. The major concern is related to the use of self-reported creativity. Many variables are already reported in the literature to predict creativity, such as personality traits, openness to experience, and age [6, 60]. Thus, in future studies, substantive control factors must be employed to isolate the effect of potential variables that may affect creativity. After controlling for potential interfering variables, potential predictors of creativity should be found.

Collecting data in Jordan involves a single data-gathering approach, thus limiting generalizability to other countries. The nonprobability convenience snowball sampling was used to collect data from universities and hospitals, restricting the generalizability of the results to other contexts. Due to the cross-sectional research design used in the current study which does not imply causation, the results should be evaluated carefully. Humble leaders involve other external or humble informal leaders, such as team mentors and coordinators, who were not studied in this research.

## 5. Summary and Conclusion

These lowest means of the studied concepts necessitate immediate organizational interventions. Quality initiatives in organizations and the number of tenures were the most effective predictors of leaders' humble leadership, participants' psychological safety, knowledge sharing in the team, and followers' creativity. Leaders' humble leadership predicted knowledge sharing in the team and followers' creativity, but not participants' psychological safety.

Leadership training and development programs are required to build nurses' and leaders' humble leadership as it contributes to followers' creativity. Workplace boundaries and supportive work environments should be established to improve participants' psychological safety pending knowledge sharing in the team, and in turn, followers' creativity occurs. Organizational attention towards creating conditions for participants' psychological safety and knowledge sharing would result in high levels of followers' creativity. Additional studies with a bigger random sample and other research designs are required in other settings and cultures, particularly those involving novel findings.

## 6. Implications

### 6.1. In Practice and Education

Leaders' humble leadership helps facilitate knowledge sharing in the team as it relates to followers' creativity of the current study's edgy participants' psychological safety, indicating that psychological safety should not warrant creative outcomes only [6], but knowledge sharing should always be present. Furthermore, workplace limits should be established so that firms can get high levels of creativity from their followers; nevertheless, psychological safety, humble leadership, and knowledge sharing should be offered concurrently [6, 12].

Also, humble leadership helps facilitate followers' creativity. Humble leadership must be acquired, developed, and sustained [6], requiring training programs to assist leaders in understanding and developing humble leadership. Humble leaders should be chosen and employed while hiring.

Since it was tense, several organizational and leadership actions effectively developed participants' psychological safety [6]. This study included encouraging followers by respecting and exploiting their unique abilities and talents and avoiding the blame culture when someone makes a mistake. These acts would help to foster supportive relationships and establish trust between humble leaders and team members [13].

Humble leadership, psychological safety, knowledge sharing, and followers' creativity are non-nursing concepts that should be integrated into the nursing curricula at the undergraduate and graduate levels, as today's students are tomorrow's nurses.

### 6.2. In Research

According to Lusso et al. [61], the researcher could only study the complete population. As a result, the conclusions derived from the created sample were intended to extend to Jordan's full population of nursing academics, nurses, nursing leaders, and organizational characteristics. Thus, the researchers should choose a sample representative of the population mentioned above.

The current results indicate that participants' psychological safety was precarious; thus, further investigation of the causes is required, including integrating qualitative research designs or mediating mechanisms to determine how concepts studied relate to one another and how they produce followers' creativity.

Like Wang et al. [6], many professionals in Jordan work in a strongly collectivist and high-power distance culture. Since humble leadership is a culturally driven notion, more research in various cultures and countries is required. Thus, more research is needed to assess leaders' humble leadership and followers' creativity.

The current study reported many novel findings, including the predictors of the concepts such as that marital status predicted leaders' humble leadership and knowledge sharing. The quality initiatives predicted participants' psychological safety and followers' creativity. These unusual findings should be examined further with bigger, randomized samples in various situations.

## Figures and Tables

**Table 1:** Subject's characteristics (*N* *=* 245).

Characteristics	^*∗*^ *N* (%)
*Gender*	
Male	76 (31.00)
Female	169 (69.00)
*Marital status*	
Single	88 (35.90)
Married	145 (59.20)
Separated or widowed	12 (4.90)
*Age*	
Less than 25 years	56 (22.90)
25–34 years	56 (22.90)
35–44 years	85 (34.70)
45–54 years	36 (14.70)
55 years or more	12 (4.90)
*Time commitment*	
Full-time work	208 (84.90)
Part-time work	37 (15.10)
*Level of education*	
Diploma (2 years college degree)	19 (7.80)
Baccalaureate	127 (51.80)
Master	58 (23.70)
Doctorate	41 (16.70)
*Role*	
Nursing academics	85 (34.70)
Nurses	140 (57.10)
Nursing leaders	20 (8.20)
*Work area*	
Faculty	57 (23.30)
Units	124 (50.60)
Wards	64 (26.10)
*Accreditation initiatives in organizations*	
Yes	219 (89.40)
No	26 (10.60)
*Quality initiatives in organizations*	
Yes	214 (87.30)
No	31 (12.70)
*Number of tenures in work*	
Less than one year	61 (24.90)
1-2 years	20 (8.20)
3-4 years	35 (14.30)
5–9 years	25 (10.20)
10 years or more	104 (42.40)
*Team size in work*	
1–5 members	63 (25.70)
6–10 members	37 (15.10)
7–15 members	42 (17.10)
More than 15 members	103 (42.00)
*The sector of work*	
Governmental	188 (76.70)
Private	57 (23.30)

^*∗*^ *N*, sample size.

**Table 2:** Levels of leaders' humble leadership (*N* *=* 245).

Items	Mean (SD^*∗*^)	Strongly disagree	Disagree	Neutral	Agree	Strongly agree
		1	2	3	4	5
This leader actively seeks feedback, even if it is critical	3.62 (1.13)	20 (8.20)	19 (7.80)	44 (18.00)	114 (46.50)	48 (19.60)
This leader admits it when they do not know how to do something	3.28 (1.14)	19 (7.80)	44 (18.00)	66 (26.90)	81 (33.10)	35 (14.30)
This leader acknowledges when others have more knowledge and skills than themselves	3.45 (1.13)	16 (6.50)	35 (14.30)	59 (24.10)	92 (37.60)	43 (17.60)
This leader takes notice of others' strengths	3.69 (1.04)	13 (5.30)	18 (7.30)	52 (21.20)	112 (45.70)	50 (20.40)
This leader often compliments others on their strengths	3.56 (1.06)	13 (5.30)	22 (9.00)	70 (28.60)	94 (38.40)	46 (18.80)
This leader shows appreciation for the unique contributions of others	3.71 (1.06)	15 (6.10)	16 (6.50)	47 (19.20)	114 (46.50)	53 (21.60)
This leader shows a willingness to learn from others	3.51 (1.13)	16 (6.50)	30 (12.20)	60 (24.50)	90 (36.70)	49 (20.00)
This leader shows that they are open to the advice of others	3.67 (1.07)	13 (5.30)	20 (8.20)	57 (23.30)	101 (41.20)	54 (22.00)
This leader shows that they are open to the ideas of others	3.72 (1.06)	14 (5.70)	14 (5.70)	56 (22.90)	103 (42.00)	58 (23.70)
Total mean score	3.57 (0.87)					

This 9-item scale is rated from 1 (strongly disagree) to 5 (strongly agree). ^*∗*^SD = standard deviation.

**Table 3:** Levels of psychological safety (*N* *=* 245).

Items	Mean (SD^*∗*^)	Strongly disagree	Disagree	Neutral	Agree	Strongly agree
		1	2	3	4	5
If you make a mistake on this team, it is often held against you	3.10 (1.05)	14 (5.70)	63 (25.70)	74 (30.20)	73 (29.80)	21 (8.60)
Members of this team can bring up problems and tough issues	3.24 (1.09)	17 (6.90)	49 (20.00)	62 (25.30)	93 (38.00)	24 (9.80)
People on this team sometimes reject others for being different	3.03 (1.13)	25 (10.20)	60 (24.50)	62 (25.30)	79 (32.20)	19 (7.80)
It is safe to take a risk on this team	3.02 (1.02)	21 (8.60)	50 (20.40)	91 (37.10)	70 (28.60)	13 (5.30)
It is difficult to ask other team members for help	2.71 (1.15)	37 (15.10)	83 (33.90)	53 (21.60)	57 (23.30)	15 (6.10)
No one on this team would deliberately act in a way that undermined my efforts	3.07 (1.01)	11 (4.50)	64 (26.10)	85 (34.70)	66 (26.90)	19 (7.80)
By working with members of this team, my unique skills and talents are valued and utilized	3.49 (0.98)	11 (4.50)	26 (10.6)	70 (28.60)	109 (44.50)	29 (11.80)
Total mean score	3.09 (0.64)					

This 7-item scale is rated from 1 (strongly disagree) to 5 (strongly agree). ^*∗*^SD = standard deviation.

**Table 4:** Levels of knowledge sharing (*N* *=* 245).

Items	Mean (SD^*∗*^)	Strongly disagree	Disagree	Neutral	Agree	Strongly agree
		1	2	3	4	5
Explicit knowledge sharing: we and our service provider share business proposals and reports with each other	3.50 (0.97)	11 (4.50)	25 (10.20)	66 (26.90)	116 (47.30)	27 (11.00)
We and our service provider share business manuals, models, and methodologies	3.44 (0.99)	12 (4.90)	29 (11.80)	67 (27.30)	112 (45.70)	25 (10.20)
We and our service provider share each other's success and failure stories	3.42 (1.01)	11 (4.50)	36 (14.70)	64 (26.10)	107 (43.70)	27 (11.00)
We and our service provider share business knowledge from newspapers, magazines, journals, and television	3.42 (0.99)	12 (4.90)	26 (10.60)	81 (33.10)	98 (40.00)	28 (11.40)
Implicit knowledge sharing: we and our service provider share know-how from work experience with each other	3.60 (0.88)	9 (3.70)	13 (5.30)	70 (28.60)	128 (52.20)	25 (10.20)
We and our service provider share each other's know-where and know-whom	3.48 (0.92)	8 (3.30)	27 (11.00)	72 (29.40)	116 (46.90)	23 (9.40)
We and our service provider share expertise obtained from education and training	3.55 (0.97)	11 (4.50)	24 (9.80)	58 (23.70)	123 (50.20)	29 (11.80)
Total mean score	3.48 (0.80)					

This 7-item scale rated from 1 (strongly disagree) to 5 (strongly agree). ^*∗*^SD = standard deviation.

**Table 5:** Levels of followers' creativity.

Items	Mean (SD^*∗*^)	Strongly disagree	Disagree	Neutral	Agree	Strongly agree
		1	2	3	4	5
I suggest new ways to achieve goals or objectives	3.81 (0.90)	8 (3.30)	10 (4.10)	49 (20.00)	131 (53.50)	47 (19.20)
I come up with new and practical ideas to improve performance	3.78 (0.84)	6 (2.40)	11 (4.50)	51 (20.80)	139 (56.70)	38 (15.50)
I search for new technologies, processes, techniques, and/or product ideas	3.73 (0.95)	8 (3.30)	13 (5.30)	65 (26.50)	110 (44.90)	49 (20.00)
I suggest new ways to increase the quality of work	3.90 (0.92)	8 (3.30)	7 (2.90)	49 (20.00)	118 (48.20)	63 (25.70)
I am a good source of creative ideas	3.80 (0.87)	5 (2.00)	9 (3.70)	64 (26.10)	118 (48.20)	49 (20.00)
I am not afraid to take risks	3.59 (0.95)	8 (3.30)	22 (9.00)	67 (27.30)	113 (46.10)	35 (14.30)
I promote and champion ideas to others	3.77 (0.85)	6 (2.40)	9 (3.70)	61 (24.90)	128 (52.20)	41 (16.70)
I exhibit creativity in my work when allowed	3.76 (0.92)	9 (3.70)	9 (3.70)	59 (24.10)	122 (49.80)	46 (18.80)
I often have new and innovative ideas	3.72 (0.81)	7 (2.90)	7 (2.90)	63 (25.70)	139 (56.70)	29 (11.80)
I come up with creative solutions to problems	3.74 (0.85)	7 (2.90)	9 (3.70)	59 (24.10)	135 (55.10)	35 (14.30)
I often have a fresh approach to problems	3.70 (0.79)	6 (2.40)	6 (2.40)	70 (28.60)	136 (55.50)	27 (11.00)
I suggest new ways of performing work tasks	3.74 (0.89)	9 (3.70)	11 (4.50)	52 (21.20)	136 (55.50)	37 (15.10)
Total mean score	3.75 (0.63)					

This 12-item scale is rated from 1 (strongly disagree) to 5 (strongly agree). ^*∗*^SD = standard deviation.

**Table 6:** Significant predictors of leaders' humble leadership, participants' psychological safety, knowledge sharing in the team, and followers' creativity based on the subject's characteristics using GLM (*N* *=* 245).

Dependent and significant predictors	*B* ^*∗*^	*T*-test	*p* value	*R* ^2^	Adjusted *R*^2^	*F*-test (df) ^*∗∗*^(*p* value)
The total score of humble leadership				0.085	0.037	16.88 (12) (0.001)
Marital status	−3.32	−2.75	0.006			
Number of tenures	3.27	1.26	0.010			
The total score of psychological safety				0.061	0.012	18.78 (12) (0.001)
Quality initiatives in organizations	−2.17	−2.37	0.018			
The total score of knowledge sharing				0.147	0.103	16.74 (12) (0.001)
Marital status	−2.41	−2.87	0.004			
Accreditation initiatives in organizations	−3.27	−2.79	0.006			
Quality initiatives in organizations	−2.44	−2.22	0.027			
Number of tenures	2.10	2.39	0.018			
The total score of followers' creativity				0.175	0.133	11.69 (12) (0.001)
Level of education	3.30	2.70	0.007			
Quality initiatives in organizations	−6.18	−3.93	0.001			
Number of tenures	2.77	2.20	0.029			

^*∗*^ *B*, unstandardized coefficients; ^*∗∗*^*p* < 0.001 (2-tailed).

**Table 7:** Significant leaders' humble leadership and subject's characteristics as predictors of participants' psychological safety, knowledge sharing in the team, and followers' creativity using GLM (*N* *=* 245).

Dependent and significant predictors	*B* ^*∗*^	*T*-test	*p* value	*R* ^2^	Adjusted *R*^2^	*F*-test (df) ^*∗∗*^(*p* value)
The total score of psychological safety				0.061	0.008	17.11 (13) (0.001)
Quality initiatives in organizations	−2.16	−2.34	0.020			
The total score of knowledge sharing				0.338	0.301	5.51 (13) (0.020)
Leaders' humble leadership	0.329	8.16	0.001			
Age	2.33	2.79	0.006			
Level of education	−2.41	−1.99	0.047			
Accreditation initiatives in organizations	−2.66	−2.55	0.011			
The total score of followers' creativity				0.325	0.287	3.15 (13) (0.077)
Leaders' humble leadership	0.425	7.16	0.001			
Level of education	3.08	2.78	0.006			
Quality initiatives in organizations	−5.12	−3.57	0.001			

^*∗*^ *B*, unstandardized coefficients; ^*∗∗*^*p* < 0.001 (2-tailed).

## Data Availability

Data are available on request due to privacy/ethical restrictions.
