# Correction to “Levels and Predictors of Leaders’ Humble Leadership, Participants’ Psychological Safety, Knowledge Sharing in the Team, and Followers’ Creativity in Nursing: A Cross‐Sectional Online Survey”

**DOI:** 10.1155/jonm/9761476

**Published:** 2026-09-25

Majd T. Mrayyan and Saleem F. Al‐Rjoub “Levels and Predictors of Leaders’ Humble Leadership, Participants’ Psychological Safety, Knowledge Sharing in the Team, and Followers’ Creativity in Nursing: A Cross‐Sectional Online Survey,” *Journal of Nursing Management*, Volume 2024, Article ID 9660787, 16 pages, 2024. https://doi.org/10.1155/2024/9660787.

While the related publication was cited in the article, this correction is provided to further clarify the relationship between the publications and to identify the specific demographic and psychological safety findings that originated from the same dataset. In both articles, the authors clearly stated the shared dataset.

Mrayyan, M. T., & Al‐Rjoub, S. (2024). Does nursing leaders’ humility leadership associate with nursing team members’ psychological safety? A cross‐sectional online survey. Journal of Advanced Nursing. https://doi.org/10.1111/jan.16117.

The authors want to clarify that the current article and the previously published article were derived from the same dataset. Although the earlier publication was appropriately cited in the original manuscript, some demographic, organizational, humble leadership, and psychological safety findings were derived from the shared dataset and were presented to provide the necessary context for the analyses reported in the current article. The inclusion of these tables was not intended to represent duplicate publication but rather to support the examination of additional research questions related to leaders’ humble leadership, knowledge sharing in teams, and followers’ creativity. Importantly, the reuse of these data does not affect the interpretation of the findings, the validity of the analyses, or the conclusions reported in the article. This correction is provided solely to further enhance transparency regarding the relationship between the two publications and the use of the shared dataset.

The previously published article was cited in the original manuscript. To further enhance transparency for readers, the following clarifications are provided.

**Methods Section**

The following statement should be added to the Methods section:

“This study was conducted using the same dataset previously reported by Mrayyan and Al‐Rjoub (2024) in the *Journal of Advanced Nursing* (https://doi.org/10.1111/jan.16117). The current article reports additional analyses from the same dataset focusing on leaders’ humble leadership, participants’ psychological safety, knowledge sharing in the team, and followers’ creativity.”

**Table 1**

The following note should be added to Table 1:

“The demographic and organizational characteristics reported in this table were derived from the same dataset previously reported by Mrayyan and Al‐Rjoub (2024) in the *Journal of Advanced Nursing.*

**Table 2**

The following note should be added to Table 2:

“The humble leadership data reported in this table were obtained from the same dataset previously reported by Mrayyan and Al‐Rjoub (2024) in the *Journal of Advanced Nursing.*

**Table 3**

The following note should be added to Table 3:

“The psychological safety data reported in this table were previously reported from the same dataset in Mrayyan and Al‐Rjoub (2024) in the *Journal of Advanced Nursing*

We apologize for this error.

**Declaration of Generative AI and AI-Assisted Technologies in the Writing Process**

During the preparation of this work, the authors used Grammarly and Copilot to edit the language. After using these tools, the authors reviewed and edited the content as needed and took full responsibility for the content of the publication.

